# Task shifting in the management of hypertension in Kinshasa, Democratic Republic of Congo: a cross-sectional study

**DOI:** 10.1186/s12913-017-2645-x

**Published:** 2017-12-04

**Authors:** Aimée M. Lulebo, Didine K. Kaba, Silvestre E.-H. Atake, Mala A. Mapatano, Eric M. Mafuta, Julien M. Mampunza, Yves Coppieters

**Affiliations:** 1 Kinshasa School of Public Health, Faculty of Medicine, University of Kinshasa, Kinshasa, Democratic Republic of the Congo; 2 Faculty of Economics and Management (FASEG), University of Lomé, Lomé, Togo; 3 Programme de santé en milieu rural (SANRU), Kongo-central, Matadi Democratic Republic of the Congo; 4 School of Public Health, Université libre de Bruxelles, Brussels, Belgium

**Keywords:** Hypertension management, Task shifting, Democratic Republic of Congo

## Abstract

**Background:**

The Democratic Republic of the Congo (DRC) is characterized by a high prevalence of hypertension (HTN) and a high proportion of uncontrolled HTN, which is indicative of poor HTN management. Effective management of HTN in the African region is challenging due to limited resources, particularly human resources for health. To address the shortage of health workers, the World Health Organization (WHO) recommends task shifting for better disease management and treatment. Although task shifting from doctors to nurses is being implemented in the DRC, there are no studies, to the best of our knowledge, that document the association between task shifting and HTN control. The aim of this study was to investigate the association between task shifting and HTN control in Kinshasa, DRC.

**Methods:**

We conducted a cross-sectional study in Kinshasa from December 2015 to January 2016 in five general referral hospitals (GRHs) and nine health centers (HCs). A total of 260 hypertensive patients participated in the study. Sociodemographic, clinical, health care costs and perceived health care quality assessment data were collected using a structured questionnaire. To examine the association between task shifting and HTN control, we assessed differences between GRH and HC patients using bivariate and multivariate analyses.

**Results:**

Almost half the patients were female (53.1%), patients’ mean age was 59.5 ± 11.4 years. Over three-fourths of patients had uncontrolled HTN. There was no significant difference in the proportion of GRH and HC patients with uncontrolled HTN (76.2% vs 77.7%, *p* = 0.771). Uncontrolled HTN was associated with co-morbidity (OR = 10.3; 95% CI: 3.8–28.3) and the type of antihypertensive drug used (OR = 4.6; 95% CI: 1.3–16.1). The mean healthcare costs in the GRHs were significantly higher than costs in the HCs (US$ 34.2 ± US$3.34 versus US$ 7.7 ± US$ 0.6, respectively).

**Conclusion:**

Uncontrolled HTN was not associated with the type of health facility. This finding suggests that the management of HTN at primary healthcare level might be just as effective as at secondary level. However, the high proportion of patients with uncontrolled HTN underscores the need for HTN management guidelines at all healthcare levels.

## Background

In 2010, 34.5 million people died from non-communicable diseases (NCDs). These deaths accounted for more than two thirds of the deaths worldwide [1]. Further, nearly 80% of these deaths occurred in low- and middle-income countries (LMICs) [2]. Hypertension (HTN) is among the main risk factors of NCDs. In 2008, the estimated prevalence of HTN in Africa was 46%, which translates to a population of nearly 30 million adults [3, 4]. Between 2003 and 2009, the WHO STEP wise approach to Surveillance (STEPS) study that was carried out in 20 African countries found that the prevalence of HTN ranged from 19.3% to 39.6% [5]. In the Democratic Republic of Congo (DRC), the prevalence of HTN was 32.1% in men and 31.5% in women in 2014 [6].

Sub-Saharan African (SSA) countries are experiencing one of the most rapid epidemiological transitions but their health systems are more oriented to the management of infectious diseases [7–9]. Effective management of HTN in SSA is challenging due to limited resources, particularly human resources for health [10, 11]. In 2009, for example, it was estimated that there were 2 physicians and 11 nurses/midwives available per 10,000 populations in SSA compared with 19 physicians and 49 nurses/midwives per 10,000 populations in North America. In 2009, in the Democratic Republic of Congo (DRC), the total number of physicians was estimated at 5827 (1 physician per 10,000 population) and the total number of nurses at 28,789 (5 nurses per 10,000 population) [12].

Task shifting has been identified as a means to address the health worker crisis and improve access and cost-effectiveness in health systems [13]. Task shifting, which dates back to the 1970s–1980s when auxiliary nurses took up the health care provider role, describes a strategy where some tasks that are normally performed by a physician or other specialized health staff are assigned to a health professional with a different or lower level of education and training, or to a person specifically trained to perform a limited task only, without having formal health education [14, 15]. Task shifting is a viable strategy in LMICs for the primary and secondary prevention of NCDs [14]. Previous studies have shown that nurses are cheaper to employ and train than doctors, Furthermore, nurses are one of the largest groups of qualified health care providers [16]. Task shifting has been shown to be cost-effective [17, 18].

Task shifting in the DRC, is based on the Primary Health Care (PHC) model. The health center (HC), which is managed by a head nurse, is the first contact between patients and health system. HCs provide accessible and affordable primary health care. Patients requiring further care are referred from the HC to a General Referral Hospital (GRH) [19]. Although a recent study carried out in HCs of Kinshasa found a low proportion of patients with controlled HTN [20], to the best of our knowledge, there have been no studies that have examined the association between task shifting and the management of HTN in the DRC. The aim of this study was to investigate the association between task shifting and the management of HTN by comparing HTN control in HCs and GRHs patients.

## Methods

### Study design

We conducted a cross-sectional study in Kinshasa Primary Health-Care (KPHC) network facilities from December 2015 to January 2016.The KPHC network consists of 51 facilities (11 GRHs and 40 HCs) that serve patients living in Kinshasa [21].

### Study population

The study included patients with an HTN diagnosis made by a healthcare provider and who were receiving care in the KPHC network facilities. Patients were eligible for the study if they were aged 18 years and older, presented for care in a participating health facilities on the day of the survey, and agreed to participate in the study.

### Sampling

The sample size was computed using the following formula:

$$ n\ge \frac{\left[p1\left(1-p1\right)+p2\left(1-p2\right)\right]}{\left(P1-P2\right)}x\left(z1-\alpha +z1-\beta \right)2 $$

Where p1 represents the proportion of patients with controlled HTN at the HC level (15.6%) [19]; p2 represents the proportion of patients with controlled HTN in the GRH level (we assumed that the proportion would be twice that in HCs (31.2%); z is the value of the standard normal distribution corresponding to a significance level of alpha of 0.05 (1.96); and β is power (80%), z1-β = 0.84.

The minimal sample size computed was 112 patients per group or 224 patients for both groups. To recruit patients, we obtained the list of GRHs and HCs from the KPHC network and visited all them to know the dates when HTN patients have follow-up clinics. We then visited the facilities on clinic dates and successively recruited patients until the desired sample size was achieved. The minimum sample size was achieved after visiting nine HCs and five GRHs.

### Data collection

Five trained data collectors’ conducted face-to-face interviews using a structured questionnaire. The questionnaire was pretested and translated into the local language (Lingala) before data collection. The questionnaire elicited information on the following variables: patients’ socio-demographic (sex, age, marital status, educational level, income) and clinical characteristics (duration of HTN, co-morbidity); health system variables (patient-provider relationship, the time devoted to consultation, waiting time, affordability and availability of healthcare); treatment related variables (types of antihypertensive drugs and dosage, experience of medication side effects, treatment adherence) and medical-related costs (consultation fees, medicines, laboratory, transportation and food).

### Study variables

The dependent variable was uncontrolled HTN, which was defined as having a systolic blood pressure (SBP) ≥ 140 mmHg and/or a diastolic blood pressure (DBP) ≥90 mmHg for patients without co-morbidity or having SBP ≥ 130 mmHg and/or DBP ≥ 80 mmHg for patients with co-morbidity [22]. The primary explanatory variable, task shifting, was defined as the management of HTN at HCs level.

Co-morbidity was defined as HTN associated with diabetes mellitus or target organ damage (heart disease, stroke, or chronic kidney disease) based on diagnosis made by a healthcare provider. Antihypertensive types were categorized in two groups, monotherapy if a patient was on one type of antihypertensive medication and multitherapy if a patient was on two or more types of antihypertensive medications. Treatment adherence was measured using the Morisky Scale, which is a validated four-item scale with good internal consistency based on studies of inner-city patients with HTN [23, 24]. Patients responded “yes” or “no” to four questions (Do you ever forget to take your medicine? Are you careless at times about taking your medicine? When you feel better, do you sometimes stop taking your medicine? Sometimes if you feel worse when you take the medicine, do you stop taking it?). Based on their responses, patients were categorized into three groups: high, medium and low adherence. To facilitate statistical analysis, adherence was dichotomized with patients with low or medium adherence classified as non-adherent and patients with high adherence classified as adherent [25].

The costs of medicines were reported on a monthly basis. The transportation costs comprised roundtrip travel costs incurred by the patient and anyone who accompanied them to the health facility for HTN-related care. Food costs comprised expenditures related to food during HTN-related health facility visits. Consultation, medicines and laboratory costs were obtained from patient record notebooks, invoices and bills provided by patients. All KPHC patients have a patient record notebook where all information related to their care including costs are recorded. Transportation and food related costs were self-reported. The cost computations did not include productivity and opportunity costs. Costs were provided in Congolese Democratic Francs (CDF), the local currency, and converted into United States Dollars (US$) using the average exchange rate during the study period (920 CDF to US$ 1). To account for inflation, a rate of 2% was used in computing all costs incurred outside the reference period (2015 to 2017).

### Statistical analysis

Data were entered into Epi data then exported to SPSS (Statistical Package for Social Sciences) version 20.0 (SPSS, Inc., Chicago, IL, USA) and Microsoft Excel for analysis. Descriptive statistics were used to summarize patients’ characteristics. Categorical variables were reported as frequencies and percentages. Continuous variables were reported using means with standard deviation. The Student’s *t* test and χ^2^ test were used to compare means and proportions respectively. Logistic regression was used to examine the association between task shifting and uncontrolled HTN after adjusting for others factors like socio-demographic, clinical factors. For all analyses, a *p*-value of less than 0.05 was considered statistically significant.

### Ethical considerations

The study protocol was reviewed and approved by the institutional review board of the Kinshasa School of Public Health. All study participants provided written informed consent.

## Results

### Patients’ socio-demographic and clinical characteristics

Patients’ socio-demographic and clinical characteristics are summarized in Table 1. Almost half of the patients were female (53.1%). The patients’ mean age was 59.5 years (standard deviation = 11.4 years). A significantly higher proportion of GRH patients than HC patients had ever attended school (87.7% vs 77.7%, *p* = 0.035). Co-morbidity was significantly more common in GRH patients compared with HC patients (45.4% vs 25.4%, *p* < 0.001). The majority (94.2%) of patients had been diagnosed with HTN within the 5 years preceding the study. Only 23% of all patients had controlled HTN. The proportion of patients with uncontrolled HTN did not differ by type of health facility (76.2% among GRH patients vs 77.7% among HC patients, *p* = 0.771).

**Table 1:** Patients’ socio demographic and clinical characteristics, by health facility

Variables	GRH patients	HC patients	Total	*p*-value
	(%) *n* = 130	(%) *n* = 130	(%) *n* = 260	
Gender				
Male	46.9	46.9	46.9	0.999
Female	53.1	53.1	53.1	
Attended school				
Yes	87.7	77.7	82.7	0.035
No	12.3	22.3	17.3	
Has a source of income				
Yes	60.8	66.2	63.5	0.371
No	39.2	33.8	36.5	
Marital status				
Married/cohabiting	72.3	67.7	70.0	0.421
Single/separated/divorced /widowed	27.7	32.3	30.0	
Co-morbidity				
Yes	45.4	25.4	35.4	<0.001
No	54.6	74.6	64.6	
HTN duration				
< 5	94.6	93.8	94.2	0.798
≥ 5	5.4	6.2	5.8	
HTN control				
Yes	23.8	22.3	23.1	0.771
No	76.2	77.7	76.9	
Mean age ± SD	58.0 ± 11.6	61.05 ± 11.0	59.5 ± 11.4	
[95% CI]	[55.9;60.1]	[59.1;62.9]	[58.1;60.9]	

*CI* confidence interval, *GRH* General Referral Hospital, *HC* Health Center, *SD* standard deviation

### Quality of health care

Patients’ assessments of the quality of care are summarized in Table 2. A significantly greater proportion of GRH patients than HC patients stated that the consultation time was sufficient (96.9% vs 81.5%, *p* < 0.001). No statistically significant differences between GRH and HC patients were observed in terms of perceived accessibility, waiting times, affordability, satisfaction with the relationship with healthcare providers, types of antihypertensive drugs used, adherence to medication, and control of HTN. A greater proportion of GRH patients than HC patients reported medication side effects (22.6% vs 8.3%, *p* = 0.003). The proportion of patients who were not adherent to medication did not differ by facility (GRH 47.2% versus HC 38.5%, *p* = 0.204). No significant difference was also found between the two groups with regard to the control of HTN.

**Table 2:** Quality of health care assessment

Variables	GRH patients	HC patients	Total	*p*-value
	(%) *n* = 130	(%) *n* = 130	(%) *N* = 260	
Time given by HCP				
Sufficient	96.9	81.5	89.2	<0.001
Insufficient	3.10	18.5	10.8	
Distance HCF-Home				
< 5 km	59.2	66.2	62.7	0.252
≥ 5 km	40.8	33.8	37.3	
Waiting time				
≤ 30 min	32.3	36.9	34.6	0.438
> 30 min	67.7	63.1	65.4	
Perception of treatment cost				
Unaffordable	63.8	70.0	66.9	0.296
Affordable	36.2	30.0	33.1	
Perception of relationship				
Very good(excellent)/good	96.9	92.3	94.6	0.109
Somewhat good/bad	3.1	7.7	5.4	
Type of anti-hypertensive				
Multitherapy	11.5	23.8	17.7	0.046
Monotherapy	70.0	60.0	65.0	0.951
No treatment	18.5	16.2	17.3	
Experience of side effects				
Yes	22.6	8.3	15.3	0.003
No	77.4	91.7	84.7	
Treatment adherence				
Yes	52.8	61.5	57.2	0.204
No	47.2	38.5	42.8	

*GRH* General Referral Hospital, *HC* Health Center, *HCP* health care provider, *HCF* health care facility

### Correlates of uncontrolled HTN

Results of the logistic regression model employed to assess the factors associated with HTN control are summarized in Table 3. The type of facility was not significantly associated with HTN control. Co-morbidity and the type of antihypertensive medication used were associated with HTN control. Patients with co-morbidity were more likely than those without co-morbidity to have uncontrolled HTN (adjusted OR = 10.3; 95% CI: 3.8–28.3) while patients on multiple medications were more likely than those on a single medication to have uncontrolled HTN (adjusted OR = 4.6; 95% CI: 1.3–16.1).

**Table 3:** Bivariate and multivariate analysis of factors associated with uncontrolled HTN

Variables	Crude OR [95%CI]	*p*-value	Adjusted OR [95%CI]^a^	*p*-value
Gender (male vs female)	1.3 [0.7–2.4]	0.353	1.3 [0.6–2.8]	0.430
Attended school				
No	0.8 [0.4–1.6]	0.530	1.7 [0.5–5.2]	0.365
Yes	1			
Co-morbidity				
Yes	8.5 [3.3–22.1]	0.000^b^	10.3 [3.8–28.3]	0.000^b^
No	1		1	
Type of anti-hypertensive drugs				
Multitherapy	4.9 [1.4–16.6]	0.011^b^	4.6 [1.3–16.1]	0.017^b^
No treatment	0.8 [0.4–1.6]	0.446	0.6 [0.3–1.3]	0.199
Monotherapy	1		1	
Experience of side effects				
Yes	1.0 [0.4–2.5]	0.965	1.1 [0.4–3.1]	0.786
No	1			
Treatment adherence				
No	0.9 [0.4–1.6]	0.658	0.9 [0.4–1.7]	0.658
Yes	1			
Time given by HCP				
Insufficient	1.1 [0.4–2.9]	0.827	0.6 [0.2–2.0]	0.390
Sufficient	1			
Type of HCF				
HC	1.1 [0.6–1.9]	0.768	0.6 [0.3–1.2]	0.160
GRH	1		1	

*CI* confidence interval, *GRH* general referral hospital, *HC* health center, *HCP* health care provider, *HCF* health care facility, *OR* odds ratio

^a^Adjusted for gender, having attended school, the time given/allocated by the provider, the experience of side effects, types of anti-hypertensive, treatment adherence and co-morbidity; ^b^ statistically significant

### Cost analysis

Table 4 shows the results of the cost analysis. In the HC, the most expensive costs were medication costs (US$4.4 [95% CI: US$3.4 – US$5.4]) while in the GRH the most expensive costs were laboratory costs (US$23.6 [95% CI: US$ 16.9 – US$ 30.3]).The mean total cost was lower in the HC than in the GRH (US$ 7.7 [95% CI: US$ 6.6 – US$ 8.8] versus US$34.2 [95% CI: US$27.6 – US$40.9]).

**Table 4:** Analysis of healthcare costs in United States Dollars (US$), by facility type

Healthcare costs	General Referral Hospital	Health Center
	Mean cost in US$ [95% CI]	Mean cost in US$ [95% CI]
Consultation costs	3.5 [2.8–4.2]	1.5 [0.3–1.8]
Medication costs	4.6 [3.8–5.3]	4.4 [3.4–5.4]
Laboratory costs	23.6 [16.9–30.3]	0.8 0.2 [0.5–1.2]
Transport costs	1.2 [1.0 – 1.5]	0.7 [0.5–0.9]
Food costs	1.4 [1.0–1.7]	0.2 [0.1–0.4]
Total costs	34.2 [27.6–40.9]	7.7 [6.6–8.8]

*CI* Confidence interval

## Discussion

In this study we investigated the association between task shifting and HTN control. Specifically, we compared patients seen in primary health centers that are managed by a head nurse and patients attending general referral hospitals who are typically managed by physicians. Similar to other studies, we found that majority of patients (76.9%) in both types of facilities had uncontrolled HTN [20, 26]. Uncontrolled HTN was not associated with the type of health facility. In contrast, Fahey and colleagues, who conducted a systematic review of randomized controlled trials (RCTs) for management of HTN, found that patients had a greater reduction in blood pressure when followed-up by non-physician health professionals [27]. However, unlike our study, Fahey’s study was based on RCTs where non-physicians were trained on management of HTN. Fahey‘s study also assessed more organizational and structural factors associated with HTN control than our study.

The presence of co-morbidity was an independent predictor of uncontrolled HTN. Studies have shown that hypertensive patients with co-morbidity have poorer blood pressure control than those without co-morbidity [28].Given this high risk, patients with co-morbidities should not be managed by primary-level care facilities that only offer a minimum package of activities/treatment [20, 29]. These results also suggest the need for guidelines on the management of HTN at the HC-level that include a global assessment of cardiovascular risk.

In contrast to other studies showing that the use of more than one antihypertensive drug is an important factor in HTN control, we found that patients on multiple antihypertensive drugs were more likely to have uncontrolled HTN than those on one drug [30, 31]. The use of more than one antihypertensive drug may mean that the patient has complicated HTN, which is difficult to control. Further, patients on multiple medications may have lower adherence [32].

Findings should be interpreted in light of the study’s limitations. First, some of the data were based on self-reports, which are subject to bias. Second, we were unable to consider additional cost components such as productivity and opportunity costs. However, our overall findings corroborated the findings of previous studies and provide useful insights on how task shifting can affect the management of HTN.

## Conclusions

This study shows that the management of HTN at primary healthcare level might be just as effective as at secondary level. However, the high proportion of patients with uncontrolled HTN underscores the need for HTN management guidelines at all healthcare levels.

## Abbreviations

DBP: Diastolic Blood Pressure
DRC: Democratic Republic of Congo
GRH: General Referral Hospital
HC: Health Center
HTN: Hypertension
KPHC: Kinshasa Primary Health-Care Network
LMICs: Low- and middle-income countries
NCDs: Non-communicable diseases
OR: Odds ratio
PHC: Primary Health Care
SBP: Systolic Blood Pressure
WHO: World Health Organization
